# Supplementary material for: Interpretable and tractable models of transcriptional noise for the rational design of single-molecule quantification experiments
Source: Nat Commun. 2022 Dec 9;13:7620. doi: 10.1038/s41467-022-34857-7 (PMC9734650; doi:10.1038/s41467-022-34857-7)
Supplement: Supplementary file 3 — Reporting Summary [file 41467_2022_34857_MOESM3_ESM.pdf]

nature portfolio

Corresponding author(s): Lior Pachter

Last updated by author(s): Oct 27, 2022

# Reporting Summary

Nature Portfolio wishes to improve the reproducibility of the work that we publish. This form provides structure for consistency and transparency in reporting. For further information on Nature Portfolio policies, see our Editorial Policies and the Editorial Policy Checklist.

## Statistics

For all statistical analyses, confirm that the following items are present in the figure legend, table legend, main text, or Methods section.

| n/a | Confirmed | |
|---|---|---|
| ☐ | ☒ | The exact sample size (*n*) for each experimental group/condition, given as a discrete number and unit of measurement |
| ☐ | ☒ | A statement on whether measurements were taken from distinct samples or whether the same sample was measured repeatedly |
| ☐ | ☒ | The statistical test(s) used AND whether they are one- or two-sided<br>*Only common tests should be described solely by name; describe more complex techniques in the Methods section.* |
| ☒ | ☐ | A description of all covariates tested |
| ☐ | ☒ | A description of any assumptions or corrections, such as tests of normality and adjustment for multiple comparisons |
| ☐ | ☒ | A full description of the statistical parameters including central tendency (e.g. means) or other basic estimates (e.g. regression coefficient) AND variation (e.g. standard deviation) or associated estimates of uncertainty (e.g. confidence intervals) |
| ☐ | ☒ | For null hypothesis testing, the test statistic (e.g. *F*, *t*, *r*) with confidence intervals, effect sizes, degrees of freedom and *P* value noted<br>*Give P values as exact values whenever suitable.* |
| ☐ | ☒ | For Bayesian analysis, information on the choice of priors and Markov chain Monte Carlo settings |
| ☒ | ☐ | For hierarchical and complex designs, identification of the appropriate level for tests and full reporting of outcomes |
| ☒ | ☐ | Estimates of effect sizes (e.g. Cohen's *d*, Pearson's *r*), indicating how they were calculated |

*Our web collection on statistics for biologists contains articles on many of the points above.*

## Software and code

Policy information about availability of computer code

| | |
|---|---|
| Data collection | New biological data were not collected for the current study. Simulated data were generated by custom routines. |
| Data analysis | scRNA-seq data were pseudoaligned using kallisto\|bustools 0.26.0, wrapping kallisto 0.46.2 and bustools 0.40.0. Dataset filtering, reduced model fits, and Akaike information criterion computation were performed using Monod 0.2.4.0. MCMC parameter inference was performed using PyMC3 3.11.4, dependent on Theano-PyMC 1.1.2. Data input/output were performed using loompy 3.0.7. Numerical procedures, such as gradient descent and quadrature, were performed using SciPy 1.4.1 and NumPy 1.21.5. The algorithms were implemented in the framework of Python 3.7.12.<br><br>All code is available at https://github.com/pachterlab/GVFP_2021 and the associated Zenodo package 10.5281/zenodo.7262328.The GitHub and Zenodo repositories include scripts used to construct a mouse genome reference, pseudoalign datasets, and generate all figures. They are modular: the analysis can be restarted at a set of intermediate steps. The outputs of certain steps, viz. pseudoaligned count matrices, results of the Monod pipeline, the list of genes of interest, results of the gradient descent procedure, and results of the Bayes factor computation procedure can be recomputed, or loaded in based on files available in the repositories.<br><br>Synthetic data generated by simulation, as well as the routines used to generate the data, are available in the repositories. The CIR simulation is implemented in Python 3.7.12. The Gamma-OU simulation was developed using MATLAB 2020a, and executed in the Python wrapper for Octave, using versions oct2py 5.4.3 and octave-kernel 0.34.1. |

For manuscripts utilizing custom algorithms or software that are central to the research but not yet described in published literature, software must be made available to editors and reviewers. We strongly encourage code deposition in a community repository (e.g. GitHub). See the Nature Portfolio guidelines for submitting code & software for further information.

## Data

Policy information about availability of data

All manuscripts must include a data availability statement. This statement should provide the following information, where applicable:

- Accession codes, unique identifiers, or web links for publicly available datasets
- A description of any restrictions on data availability
- For clinical datasets or third party data, please ensure that the statement adheres to our policy

Publicly available data were downloaded from the NeMO archive. The metadata were obtained from http://data.nemoarchive.org/biccn/grant/u19_zeng/zeng/transcriptome/scell/10x_v3/mouse/processed/analysis/10X_cells_v3_AIBS/. Raw FASTQs were obtained from http://data.nemoarchive.org/biccn/grant/u19_zeng/zeng/transcriptome/scell/10x_v3/mouse/raw/MOp/. Pre-built genome references were obtained from the 10x Genomics website, at https://support.10xgenomics.com/single-cell-gene-expression/software/downloads/latest.

The FASTQ files were used to generate loom files with spliced and unspliced count matrices. These count matrices are available in the Zenodo package 10.5281/zenodo.7262328. The results of the fits generated with the Monod package, the SDE gradient descent fit, and the MCMC fit are available at https://github.com/pachterlab/GVFP_2021, as well as the Zenodo package 10.5281/zenodo.7262328.

All synthetic data, generated using custom stochastic simulation code, as well as the simulation parameters, are deposited in the GitHub and Zenodo repositories.

# Field-specific reporting

Please select the one below that is the best fit for your research. If you are not sure, read the appropriate sections before making your selection.

☒ Life sciences ☐ Behavioural & social sciences ☐ Ecological, evolutionary & environmental sciences

For a reference copy of the document with all sections, see nature.com/documents/nr-reporting-summary-flat.pdf

# Life sciences study design

All studies must disclose on these points even when the disclosure is negative.

| | |
|---|---|
| Sample size | scRNA-seq data analysis used data from 4 mice. Monod gene filtering used a subset of these data, 5 glutamatergic cell subtype populations with 251-2395 cells per subtype, taken from a single mouse. SDE model fits used aggregated glutamatergic cell populations, with 4497-6604 cells per sample, from all 4 mouse samples. These cell population samples were based on existing annotations, released along with doi:10.1038/s41586-021-03969-3, adopted to limit potential heterogeneity due to differences among cell types rather than biological variation. The analyzed samples were constructed by extracting cell barcodes from existing annotations, and omitting 0-69 cells fewer than 10,000 spliced RNA counts. The sample sizes are given in Supplementary Table 6.<br><br>Simulation benchmarking used 10,000 simulated cells per condition, set relatively high to illustrate the numerical concordance between simulations and analytical solutions.<br><br>Inference from simulated data used 100-5000 simulated cells per condition. These sample sizes are comparable to cell type population counts in typical single-cell RNA sequencing experiments, and consistent with the (251 to 6604)-cell populations analyzed in the mouse brain datasets. The range of simulated sample sizes was chosen to provide a realistic benchmark for inference from real data and characterize theoretical performance for putatively homogeneous cell types. |
| Data exclusions | For real data inference, cells with fewer than 10,000 RNA counts were excluded from analysis, as they are typically considered to be "empty droplets." The knee plot used to motivate this filtering is given in Supplementary Fig. 7. Cells outside the glutamatergic cell types were excluded to attempt to match the model's assumption of homogeneity. Cells in the small L6 IT Car3 and L5 ET subtypes were excluded to allow the interpretation of the B08 glutamatergic dataset as the union of the five subtypes analyzed in the gene filtering step. Genes that were not consistently assigned to a particular reduced model, as quantified by the Akaike information criterion, were excluded from further analysis, due to our interest in exploring the bounds of applicability and discriminability of the introduced theoretical models in real data. Only the top 35 genes in each category of interest, corresponding to those achieving the lowest (chi-squared) distance between fit and data. Other genes were excluded to avoid potential contributions of model misspecification and suboptimal gradient descent convergence. After SDE fits, genes with absolute log-likelihood ratio exceeding 150 were excluded from visualization. As shown in Supplementary Figs. 8-37, the genes with higher log-LRs appear to have converged to suboptimal estimates of the maximum likelihood parameters. |
| Replication | Glutamatergic cell type datasets from four distinct mice were used. To ensure inference replication on MCMC, we used four independent chains. To ensure likelihood ratio inference replication, we used 15 independent starts for gradient descent. All analyses were performed in Jupyter notebooks and can be replicated using the code deposited on GitHub and in Zenodo. |
| Randomization | As the analysis took place on publicly available data, and we selected similar tissues for replication and comparison, randomization was not used. |
| Blinding | The majority of our work is an exploratory model analysis, where we derive theoretical solutions and identifiability results, so blinding is not pertinent. We did perform data analysis to test model fit based on the derived theoretical results. In this setting, blinding was not relevant, as we were assessing model fit and qualitative predictive value, rather than associating an intervention with outcome. The fit and analysis procedures were identical for all genes, reducing potential bias in the results. |

# Reporting for specific materials, systems and methods

We require information from authors about some types of materials, experimental systems and methods used in many studies. Here, indicate whether each material, system or method listed is relevant to your study. If you are not sure if a list item applies to your research, read the appropriate section before selecting a response.

## Materials & experimental systems

| n/a | Involved in the study |
|---|---|
| ☒ | ☐ Antibodies |
| ☒ | ☐ Eukaryotic cell lines |
| ☒ | ☐ Palaeontology and archaeology |
| ☒ | ☐ Animals and other organisms |
| ☒ | ☐ Human research participants |
| ☒ | ☐ Clinical data |
| ☒ | ☐ Dual use research of concern |

## Methods

| n/a | Involved in the study |
|---|---|
| ☒ | ☐ ChIP-seq |
| ☒ | ☐ Flow cytometry |
| ☒ | ☐ MRI-based neuroimaging |



March 2021
